# Immunogenicity of antigens from the TbD1 region present in *M. africanum *and missing from "modern" *M. tuberculosis*: a cross- sectional study

**DOI:** 10.1186/1471-2334-10-11

**Published:** 2010-01-19

**Authors:** Bouke C de Jong, Abdulrahman Hammond, Jacob K Otu, Martin Antonio, Richard A Adegbola, Martin O Ota

**Affiliations:** 1 Bacterial Diseases Programme, MRC Laboratories, POB 273, Banjul, the Gambia; 2 New York University, 550 First Avenue, Smilow 901, New York, NY 10016, USA; 3 FMPOS, University of Bamako, Point G, Bamako, Mali

## Abstract

**Background:**

Currently available tools cannot be used to distinguish between sub-species of the *M. tuberculosis *complex causing latent tuberculosis (TB) infection. *M. africanum *causes up to half of TB in West- Africa and its relatively lower progression to disease suggests the presence of a large reservoir of latent infection relative to *M. tuberculosis*.

**Methods:**

We assessed the immunogenicity of the TbD1 region, present in *M. africanum *and absent from "modern" *M. tuberculosis*, in an ELISPOT assay using cells from confirmed *M. africanum *or *M. tuberculosis *infected TB patients without HIV infection in the Gambia.

**Results:**

Antigens from the TbD1 region induced IFNγ responses in only 35% patients and did not discriminate between patients infected with *M. africanum *vs. *M. tuberculosis*, while PPD induced universally high responses.

**Conclusions:**

Further studies will need to assess other antigens unique to *M. africanum *that may induce discriminatory immune responses.

## Background

An estimated third of the world's population is latently (asymptomatically) infected with *M. tuberculosis*, of whom 5-10% will progress to TB disease in their lifetime, with higher rates of progression in immune compromised people [1]. Latent infection can be diagnosed with the tuberculin skin test, or more recently with interferon gamma release assays using *M. tuberculosis *specific antigens ESAT-6 and CFP-10 [2]. These antigens are present in members of the *M. tuberculosis *complex, except for the vaccine strain *M. bovis *BCG [3], *M. microti *and the Dassie bacillus.

Recent data suggest that *M. africanum *has a relatively large reservoir of latent infection that supports the 38% prevalence of active disease despite a lower rate of progression.

Better characterization of the immune responses leading either to containment or progression of latent TB infection to disease by *M. africanum *or *M. tuberculosis *requires diagnosis of these organisms at the latent stage of infection. During latent TB infection, the bacterial load is estimated to be low, and the location of the persistent bacteria is unknown. Molecular genotyping methods rely on the isolation of bacterial DNA, which is currently not possible from patients with latent TB infection. Nevertheless, the persistent bacteria induce an immune response that can be assessed in the periphery [1].

The TbD1 region is present in *M. africanum *but absent in "modern" *M. tuberculosis *[3]. *M. bovis *has not yet been isolated from humans or bovines in the Gambia [4,5], and "ancient" *M. tuberculosis *causes only 3.4% of TB in the Gambia [6].

Therefore we hypothesized that a response to TbD1 antigens should be relatively specific for *M. africanum*, which could subsequently be used to determine the prevalence of latent infection with *M. africanum*. We tested the sensitivity and specificity of these TbD1 antigens in an ex-vivo IFN-γ ELISPOT assay in TB cases infected with *M. africanum *or *M. tuberculosis*.

## Methods

After informed consent, adult patients with smear positive TB were recruited in an ongoing TB Case Contact study in the Gambia and offered Voluntary Counseling and Testing for HIV as described previously [2]. This study was approved by the joint Gambian Government/MRC ethics committee and by the IRB from New York University. HIV infected patients were excluded from this analysis.

### Synthetic peptides

Antigens were prepared from TbD1, a region missing from "modern" *M. tuberculosis *and present in *M. africanum *and *M. bovis *[3]. TbD1 contains 2 genes, mmpL6 and mmpS6, of which mmpL6 was prepared as 2 separate peptides, mmpL6-C and mmpl6-N. Due to constraints in the number of cells, we initially screened these two peptides from mmpL6 in separate wells in ELISPOT, and later selected mmpL6-C together with mmpS6 as test antigens.

### RD-1 and TbD1 antigens in ex vivo IFNγ ELISPOT

We used an *ex vivo *ELISPOT technique to enumerate frequencies of circulatory IFNγ -producing T cell responses as shown previously [2]. Briefly, peripheral blood mononuclear cells (PBMC) were isolated from 15 ml heparinized whole blood and resuspended in RPMI-1640 culture media supplemented with100 IU/ml penicillin, 100 μg/ml streptomycin, 5% heat-inactivated AB human serum (Sigma), and 2 mM L-glutamine. The cells were then plated in duplicate at 2 × 10^5 ^per well, and cultured for 18 h at 37°C with the following antigens: medium alone, ESAT-6 peptide pool (EP, 2.5 μg/ml), CFP-10 peptide pool (CP, 2.5 μg/ml) [2,7,8], ESAT-6/CFP-10 fusion protein (FP, 5 μg/ml), PPD-tuberculin (PPD-T [*M. tuberculosis*, RT49; Statens serum Institut, Copenhagen, Denmark], 20 μg/ml), peptides corresponding to TbD1 antigen (prepared by C. Franken, 10 μg/ml), and PHA (Sigma-Aldrich, 5 μg/ml) that served as a positive control. The ELISPOT plates were developed following the manufacturer's instructions and spot forming units (SFU) were scored with an automated ELISPOT counter (AID-GmBH, Strasbourg, Germany). A positive response (cut-off) was recorded if SFU were ≥ 8 spots above background and there were twice as many spots as negative control wells. Data were excluded if PHA positive control wells were <150 spots [7-9].

### Laboratory isolates and genotyping

All TB cases submitted sputum for conventional mycobacteriology, including Acid Fast smears and cultures in liquid and solid media as described previously [6]. By genotyping the isolates from these patients using spoligotyping [10], we classified the patients as infected with *M. africanum *or *M. tuberculosis *[5].

### Statistical analysis

We used Stata software (version 10; Stata-Corp) and performed the non-parametric Mann-Whitney test to compare continuous variables, considering *P *< 0.05 as statistically significant.

## Results

### Patient and isolate characteristics

We enrolled 81 TB patients, of whom 74 (91%) agreed with HIV testing, including nine who tested positive (12.2%, eight with HIV-1 and one with HIV-2). Of the 65 HIV negative TB patients, one patient had uninterpretable ELISPOT results, two did not have a genotyped bacterial isolate, and 62 (77%) patients were included in the analysis. Both mmpL6-C and mmpL6-N peptides were tested in 24 patients, while mmpL6-C peptide alone was tested in combination with the mmpS6 peptide in 38 patients. TB disease was caused by *M. africanum *in 21 (34%) of the patients, with the remainder due to *M. tuberculosis*, including 3 (4.8%) "ancient" *M. tuberculosis *isolates with intact TbD1 region.

### Immunogenicity of *M. africanum *specific peptides

**Figure 1:**
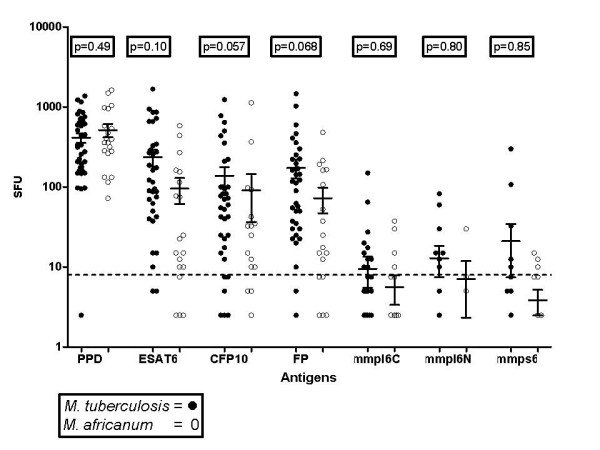
**Differences in ELISPOT responses between TB casesinfected with *M. tuberculosis *versus *M. africanum***. The TbD1 based antigens mmpl6C, mmpl6N, and mmps6 did not induce robust responses in *M. africanum *infected patients, despite the presence of an intact TbD1 region in *M. africanum*. The horizontal lines indicate the mean and SEM. FP = ESAT-6/CFP-10 fusion protein.

The mmpL6-C peptide induced IFNγ responses in 19% of *M. africanum *infected cases, the mmpL6-N peptide in 17%, and the mmpS6 peptide in 20%. The proportion who responded to any of the three TbD1 antigens was similar between *M. africanum *infected patients and *M. tuberculosis *infected patients (respectively 33% and 37%, p = 0.80). Equal numbers of patients infected with *M. africanum *and with *M. tuberculosis *responded to PPD (respectively 95% and 90%, p = 0.49), whereas responses to ESAT-6, CFP-10 and ESAT-6/CFP-10 fusion protein tended to be lower in *M. africanum *infected patients (Figure 1).

Of the three patients infected with "ancient" *M. tuberculosis*, one responded to TbD1 antigens and two did not. The results did not change significantly with the inclusion or exclusion of these cases as *M. tuberculosis*-infected in the analysis.

## Discussion

To our knowledge, this study is the first attempt at uncovering TB sub-species specific T cell responses in the natural human host. Our findings suggest that the antigens prepared from TbD1 do not induce a robust T cell response in humans. Among the responders, there was moreover no difference in TbD1 recognition between the proportion of patients infected with "modern" *M. tuberculosis*, which lacks the TbD1 region, and the proportion infected with *M. africanum*, with an intact TbD1 region. Lastly, we identified a trend towards attenuated immunogenicity of ESAT-6 and CFP-10 in *M. africanum *infected TB cases, on which we reported previously [11].

The low proportion of responders may be due to poor host recognition of these antigens, or due to the fact that the host response includes cytokines other than IFNγ or B cell rather than T cell responses. Indeed, mmpL6 is a putative transmembrane transport protein [12] and may induce a B cell response against its extracellular tail.

Although we did not identify instances of dual infection from the cultured sputum samples, the presence of dual infection could explain the inability of the TbD1 antigens to discriminate between those diseased with *M. africanum *from *M. tuberculosis*. Undetected dual infection in TB patients could occur when one organism was maintained in latency while the other caused TB disease, or when both organisms partook in active TB disease, but one organism outgrew the other in culture. The latter can be addressed by genotyping the isolates with species-specific primers using DNA extracted from sputum samples.

The *M. africanum *whole genome sequencing project is near completion, which will facilitate the search for other discriminatory antigens unique to *M. africanum*. The TbD1 region, shared by *M. africanum *and *M. bovis*, is the only region identified in the *M. bovis *genome that is absent from *M. tuberculosis*, so our findings may also inform veterinary research directed at distinguishing *M. bovis*- vs "modern" *M. tuberculosis *specific responses in latently infected cows.

## Conclusions

In conclusion, assessment of the prevalence of TB latency with one or more different sub-species within the *M. tuberculosis *complex will depend on the detection of specific host responses, due to inability to extract mycobacterial DNA from latently infected individuals. The TbD1 region, which is absent in "modern" *M. tuberculosis *and present in *M. africanum *and *M. bovis*, does not induce a discriminatory IFNγ T cell response in TB patients in the Gambia. Further studies will need to assess whether TbD1 induces B cell responses, which would facilitate assessment of the prevalence of latent infection with distinct sub-species. Alternatively, the whole genome sequence may reveal other antigens unique to *M. africanum *that may induce T cell- and/or B cell responses.

## Competing interests

The authors declare that they have no competing interests.

## Authors' contributions

B CJ conceived of the study, carried out the genotyping studies, performed the statistical analysis and drafted the manuscript. AH carried out the immunoassays and helped to draft the manuscript. JO conducted the mycobacterial studies. MA coordinated mycobacterial studies and molecular analysis. RAA participated in study design and coordination. MOO participated in study design and coordination and helped to draft the manuscript. All authors read and approved the final manuscript.

## Pre-publication history

The pre-publication history for this paper can be accessed here:

http://www.biomedcentral.com/1471-2334/10/11/prepub
